# Perioperative Management of Patients with Diabetes and Cancer: Challenges and Opportunities

**DOI:** 10.3390/cancers16162821

**Published:** 2024-08-11

**Authors:** Mohamed Shouman, Michelle Brabant, Noor Rehman, Shahid Ahmed, Rabia K. Shahid

**Affiliations:** 1 Saskatchewan Cancer Agency, Regina, SK S4W 0G3, Canada; 2 Department of Oncology, University of Saskatchewan, Saskatoon, SK S7N 4H4, Canada; 3 Department of Medical Oncology, National Cancer Institute, Cairo 11796, Egypt; 4 Department of Medicine, University of Saskatchewan, Saskatoon, SK S7N 5E5, Canada; 5 College of Medicine, University of Saskatchewan, Saskatoon, SK S7N 5E5, Canada

**Keywords:** diabetes mellitus, cancer, surgery, operative complications, surgical procedure, perioperative care, diabetes complications

## Abstract

**Simple Summary:**

Diabetes and cancer are major global health problems that cause significant illness and death. Many cancer patients also have diabetes, which complicates their treatment, especially if surgery is needed. This review highlights the link between diabetes and cancer. It looks into how cancer treatments can worsen blood sugar control, examines the risks that surgery holds for patients with both conditions, and outlines the strategies that can be used to manage diabetes around the time of surgery. More research is needed to provide new strategies that can reduce complications following surgery in patients with diabetes and cancer.

**Abstract:**

Background: Both diabetes and cancer are major global health issues that are among the leading causes of morbidity and mortality. There is a high prevalence of diabetes among cancer patients, many of whom require a surgical procedure. This review focuses on the operative complications in patients with diabetes and cancer, and the perioperative management of diabetes in cancer patients. Methodology: A literature search of articles in English—published between January 2010 and May 2024—was carried out using the databases PubMed, MEDLINE, Google Scholar, and the Cochrane Database of Systematic Reviews. The search primarily focused on the operative complications in patients with diabetes and cancer, and perioperative management strategies. Results: The relationship between cancer and diabetes is complex; cancer patients have a high risk of developing diabetes, while diabetes is a risk factor for certain cancers. In addition, various cancer therapies can induce or worsen diabetes in susceptible patients. Many individuals with cancer and diabetes require surgery, and due to underlying diabetes, they may have elevated risks for operative complications. Optimal perioperative management for these patients includes managing perioperative glycemia and other comorbid illnesses, adjusting diabetic and cancer treatments, optimizing nutrition, minimizing the duration of fasting, supporting early mobilization, and providing patient education to enable self-management. Conclusions: While evidence is limited, optimal perioperative management for patients with both diabetes and cancer is necessary in order to reduce surgical complications. Future studies are needed to develop evidence-informed perioperative strategies and improve outcomes for these patients.

## 1. Introduction

Diabetes is a complex chronic condition that affects millions of individuals worldwide [1]. The Global Burden of Disease Collaborator estimates that around 6% of the world’s population is affected by diabetes, meaning that more than half a billion people are currently living with diabetes [2]. Approximately 25 to 30% of people with diabetes first become aware of having diabetes when disease-related complications arise [1,2,3]. Patients with diabetes are at high risk of mortality from acute and chronic complications related to vascular damage [4,5]. In addition, overall mortality rates are up to five times higher in diabetic patients compared to non-diabetic patients. Moreover, there is a strong association between diabetes and cancer, and diabetes is more prevalent in patients with cancer. Diabetes is not only a risk factor for several malignancies, but has also been associated with inferior outcomes [5]. Furthermore, several medications that are used in cancer care are associated with high risks for hyperglycemia and diabetes development.

**Figure 1:**
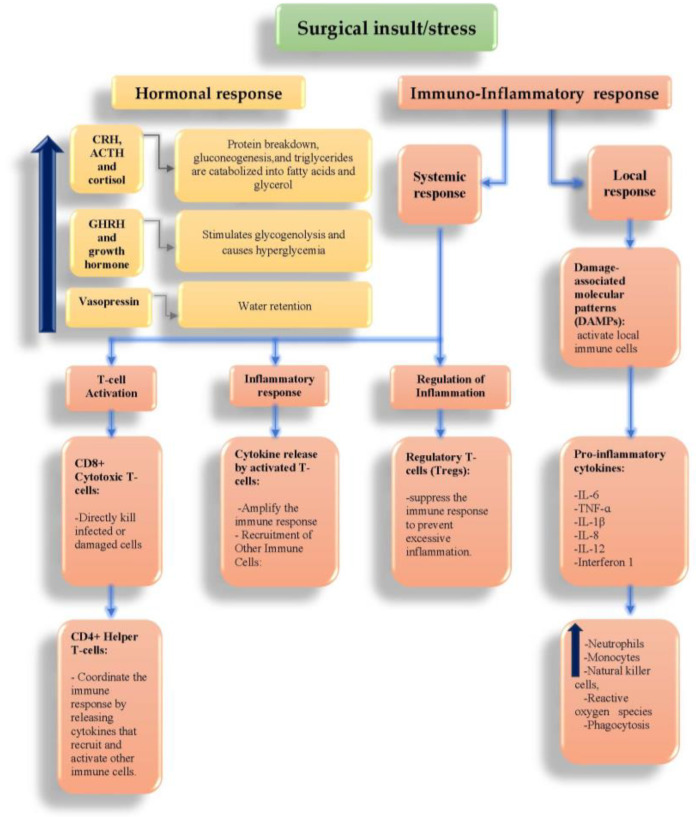
Hormonal and inflammatory-immune responses to surgery. CRH, corticotrophin-releasing hormone; ACTH, adrenocorticotropic hormone; GHRH, growth hormone-releasing hormone; IL, interleukin; TNF-ɑ, tumor necrosis factor-alpha; CD, cluster of differentiation; Th1/Th2, type 1/type 2 T helper cells; HMGB1, high-mobility group box 1; ATP, adenosine triphosphate; HSPs, heat shock proteins.

Surgery remains the primary curative treatment for the majority of early-stage solid organ cancers. Certain oncologic surgeries, such as pancreaticoduodenectomy or esophagectomy, are notably complex and lengthy procedures that carry a high risk of complications, including mortality [6]. In contrast, other procedures such as excision biopsy of suspicious lesions are short and entail limited risks. Additionally, individuals with both diabetes and cancer commonly need non-oncologic surgeries, such as knee joint replacements. Approximately 20% of patients undergoing surgery have diabetes, which is associated with up to a 50% higher risk of surgical morbidity or mortality compared to those without diabetes [4,7]. Furthermore, patients with diabetes undergo more surgeries than those without diabetes [8]. Surgery induces stress on the body, leading to alterations in various physiological responses; these include changes in inflammatory and acute-phase responses, as well as hormonal responses such as the secretion of adrenocorticotropin and growth hormone, which can cause hyperglycemia [9,10] (Figure 1).

The optimal management of blood glucose—which involves comprehensive preoperative assessment and glycemic control, intraoperative glucose management, and postoperative diabetes care—is vital for reducing perioperative complications, averting delays in commencing adjuvant therapy, and minimizing toxicities associated with subsequent cancer treatments such as systemic therapy and radiation. However, there is a dearth of literature studies focusing on the perioperative management of diabetes in cancer patients.

This paper aims to review the existing literature on the perioperative management of diabetes in cancer patients. It starts by examining the epidemiology of cancer and diabetes, the common cancer medications that cause hyperglycemia, and the complications of surgery in diabetic patients, and it goes on to focus on perioperative diabetes management in cancer patients and potential areas for future research.

## 2. Methodology

A literature review was conducted using a range of terms deemed relevant to this review. Due to the very broad scope of this paper, a systematic review of the literature was not feasible. Searches were carried out within various databases, including PubMed, MEDLINE, Google Scholar, and the Cochrane Database of Systematic Reviews. In addition, pertinent proceedings and guidelines from specialty societies were reviewed. Most searches covered the period from January 2010 to May 2024; however, certain topics needed an extended search from 1980 to May 2024. Only articles in English were captured in the searches. Given that this review explores various aspects of perioperative management of patients with both cancer and diabetes mellitus, we conducted multiple independent searches using a range of keywords combined with “diabetes,” “cancer,” with or without “perioperative care” to identify key papers pertinent to each section. Priority was given to meta-analyses, systematic reviews of observational studies, pooled analyses of clinical trials, or large multicenter cohort studies where available. In sections with limited evidence, smaller cohort studies were included.

## 3. Epidemiology

Cancer presents a significant global challenge; in 2022, an estimated 20 million new cases were diagnosed and approximately 10 million cancer deaths were reported in the same year. The anticipated global burden of cancer is projected to reach 28.4 million cases by 2040, reflecting a greater than 40% increase [11]. Diabetes is another major health concern, and there are currently more than 500 million adults living with diabetes around the world. By 2040, it is projected that the number of individuals aged 20–79 years with diabetes will increase to 642 million worldwide [12]. The prevalence of diabetes in cancer patients varies, ranging from 10% to 30% or higher, depending on various factors such as underlying cancer, patient demographics, and geographical location [5,13]. There are strong associations between type 2 diabetes mellitus (T2DM) and colorectal, hepatocellular, gallbladder, breast, endometrial, and pancreatic cancers [14,15]. Although the exact mechanisms behind these associations are unclear, it is thought that oxidative stress, hyperinsulinemia, genetic predisposition, and inflammation could be linked to the development or progression of some types of malignancies [5,16]. Pre-existing diabetes or hyperglycemia in cancer patients independently increases the risk of mortality [17,18]. A systematic review and meta-analysis of 23 studies revealed that individuals with diabetes had a 41% higher risk of mortality (hazard ratio [HR] = 1.41, 95% confidence interval [CI] = 1.28–1.55) compared to those with normal blood glucose levels, across various cancer types [17]. Additionally, a meta-analysis of 12 observational studies involving 9872 cancer patients without diabetes found that individuals with hyperglycemia showed significantly worse disease-free survival (HR = 1.98, 95% confidence interval [CI] = 1.20–3.27) compared to those with normoglycemia, as well as poorer overall survival (HR 2.05, 95% CI 1.67–2.55) [18].

## 4. Classification of Diabetes

Diabetes mellitus is classified into several groups [19]. Type 1 diabetes results from immune-mediated damage to the beta cells of the pancreas, leading to absolute insulin deficiency. In contrast, type 2 diabetes arises from varying degrees of insulin resistance and deficiency due to several environmental and genetic factors such as obesity, which results in hyperglycemia. Type 2 diabetes is the most common form of diabetes. Maturity-onset diabetes in young people is characterized by an early onset of hyperglycemia, typically before the age of 25 years, and it is associated with impaired insulin secretion with little or no disruption in insulin function. Other types of diabetes are known as secondary diabetes. Among these, pancreatic diabetes—which is also called type 3c diabetes—results from the structural and functional impairment of insulin secretion from the pancreatic beta cells due to dysfunction of the exocrine pancreas from cancer, pancreatitis, cystic fibrosis, trauma, hemochromatosis, and rare genetic disorders. In addition, drug-induced diabetes is relatively common in cancer patients; it is particularly related to glucocorticoids, targeted therapy, and immune checkpoint inhibitors [20]. Overall, type 2 diabetes mellitus is the most prevalent type in cancer patients [14].

## 5. Hyperglycemia Caused by Antineoplastic Agents

Systemic therapy have evolved significantly over the past few decades, but they still come with many adverse effects, including metabolic disturbances such as hyperglycemia. Hyperglycemia in cancer patients can arise from various causes, including risk factors shared with diabetes such as older age, male sex, obesity, and lifestyle choices such as a lack of physical activity, a high-calorie diet, and smoking [21]. These factors, combined with acute stress from cytotoxic and targeted agents, can exacerbate insulin resistance and lead to hyperglycemia. Hyperglycemia can occur in 10–30% of patients during chemotherapy [21]. It can complicate cancer treatment and increase the likelihood of additional health problems, such as infections, delayed wound healing, and exacerbated symptoms. Understanding which of the relevant antineoplastic agents are likely to cause hyperglycemia is crucial for managing and mitigating perioperative complications in cancer patients with diabetes. 

Various cancer therapies can induce or worsen diabetes in susceptible patients. These include glucocorticoids, antimetabolites like 5-fluorouracil and decitabine/azacitidine, PI3K/AKT/mTOR inhibitors, ALK inhibitors such as ceritinib, certain tyrosine kinase inhibitors like nilotinib, octreotide, and immune checkpoint inhibitors [22,23]. The following section focuses on commonly used cancer medications that are associated with hyperglycemia and the exacerbation of diabetes.

### 5.1. Corticosteroids

Corticosteroids are often used in combination with chemotherapy to control nausea, vomiting, and to reduce chemotherapy-related allergic reactions. They are also a key component in the treatment protocol for certain hematological cancers, such as myeloma. Glucocorticoids can cause insulin resistance and hyperglycemia by reducing the uptake of glucose by muscle and adipose tissue, increasing hepatic gluconeogenesis, and preventing glucose uptake into peripheral tissues [21,24]. 

Several risk factors contribute to the likelihood of patients developing glucocorticoid-related hyperglycemia, such as the dose and duration of glucocorticoid treatment, older age, higher weight, a family history of diabetes, and non-white ethnicity [24]. Over 50% of patients without a known history of diabetes who receive glucocorticoids may develop transient hyperglycemia [21]. 

### 5.2. Targeted Therapies: PI3K/AKT/mTOR Pathway

Among the targeted therapies, the PI3K/AKT/mTOR pathway inhibitors are well known to cause hyperglycemia. mTOR inhibitors such as everolimus and temsirolimus disrupt the mTOR signaling pathway, impairing glucose homeostasis by inhibiting glycogen synthesis and promoting glycolysis, which leads to increased blood glucose levels [25,26]. They also cause peripheral insulin resistance by reducing glucose uptake by muscle mass. Clinical trials have reported a 13–50% prevalence of new-onset diabetes and hyperglycemia in people taking mTOR inhibitors [21,27]. Notably, the majority of significant hyperglycemic events in these trials, classified as grade 2 or higher, tend to occur within the first six weeks of treatment [21].

KT/PI3K pathway inhibitors such as alpelisib, idelalisib, and capivasertib inhibit insulin’s action, leading to decreased glucose uptake by cells and increased glucose production by the liver, thereby resulting in elevated blood glucose levels and insulin resistance [28]. Clinical trials have reported high rates of hyperglycemia in patients treated with AKT/PI3K/mTOR inhibitors, necessitating careful monitoring and management of blood sugar levels in these patients when they are undergoing surgery [26].

### 5.3. Immune Checkpoint Inhibitors

Immune checkpoint inhibitors (ICIs) enhance the immune system’s ability to target cancer cells by blocking inhibitory checkpoints such as PD-1 and CTLA-4. However, they can cause autoimmune side effects in addition to hyperglycemia, which occurs in about 9% of patients with no prior history of diabetes [29]. ICI-induced diabetes—which resembles type 1 diabetes caused by the autoimmune destruction of pancreatic beta cells—often presents as diabetic ketoacidosis. Additionally, ICIs can lead to other autoimmune conditions requiring high-dose glucocorticoids, which further contribute to hyperglycemia.

Monitoring blood glucose levels in non-diabetic patients receiving medications that could cause hyperglycemia and undergoing surgery is crucial to avoid surgical complications. Regular blood glucose monitoring helps prevent complications from untreated hyperglycemia.

## 6. Operative Complications in Diabetes

**Figure 2:**
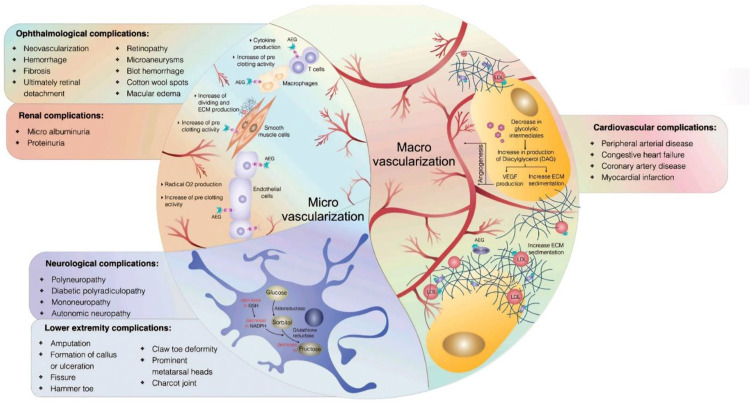
Microvascular and macrovascular complications associated with diabetes mellitus. Reproduced with permission from Saberzadeh-Ardestani B et al. *Cell J.* 2018 [31].

Diabetes is well known to cause a wide range of complications; more than a third of diabetic patients are expected to have microvascular or macrovascular complications that result in organ damage [30] (Figure 2). 

The course of diabetes can be complicated by other life-threatening metabolic conditions such as diabetic ketoacidosis, hyperglycemic hyperosmolar syndrome, or hypoglycemia, which may occur during the perioperative period, and be aggravated by surgery-induced stress, anesthesia, infections, and diet [32].

Approximately 80% of individuals diagnosed with cancer will undergo some type of surgical procedure as part of their treatment regimen [33]. These surgeries encompass a range of interventions, from low-risk procedures such as biopsies and porta Cath placements for chemotherapy administration to major tumor resections. Surgery can increase blood glucose levels due to the release of catecholamines and cortisol and an overactive sympathetic nervous system, which reduces insulin sensitivity. This can lead to elevated glucagon and growth hormone levels, ultimately disturbing glucose homeostasis [34].

A prospective observational study involving 7565 patients showed that, compared to non-diabetic patients, diabetic patients had 29% higher 6-month postoperative mortality, higher ICU admission (27% in diabetics vs. 18% in non-diabetics), higher ventilation rates (16% in diabetics vs. 10% in non-diabetics), and longer hospital stays [35]. Another prospective observational study of 3201 patients undergoing coronary artery bypass surgery found that higher glycated hemoglobin (HbA1c) levels (>7%) were associated with reduced long-term survival. For each unit increase in HbA1c, there was a significant 15% reduction in 5-year survival. Among diabetic patients treated with insulin, 5-year survival was significantly worse compared with patients treated with diet, oral hypoglycemic medication, or nothing (78.3% vs. 82.4%) [36]. Similarly, other studies have shown that patients treated with insulin had poorer postoperative outcomes compared to those treated with oral hypoglycemic medication [37,38].

Cancer patients with diabetes have a high likelihood of postoperative complications and mortality. A meta-analysis conducted by Barone and colleagues, which included 23 studies, found that cancer patients with pre-existing diabetes had a higher risk of all-cause mortality compared to non-diabetic cancer patients, with a hazard ratio (HR) of 1.41 (95% [CI = 1.28–1.55]) [17]. In another systematic review and meta-analysis of 20 studies, the postoperative mortality rate was 50% higher in diabetic patients with various cancers compared to normoglycemic patients, with a median 30-day post-surgery mortality of 6.5% [39]. Older studies have shown significantly higher short-term mortality in diabetic patients with colon cancer compared to non-diabetic patients undergoing either emergency or scheduled resection surgery [40,41,42,43]. A recent systematic review and meta-analysis of 55 studies involving 666,886 patients found that diabetic patients with colorectal cancer have higher rates of postoperative complications, including anastomotic leaks and hospital readmissions, compared to non-diabetic patients [44].

Some studies have shown that stress-induced hyperglycemia, regardless of diabetic status, is associated with poorer mortality outcomes [45,46,47]. Additionally, elevated postoperative glucose levels, independent of preoperative diabetic status, were associated with a 30% higher infection risk for every 2.2 mmol/L increase above 6.1 mmol/L, as well as prolonged hospital stays [48]. Diabetic patients often have several underlying comorbidities, such as nephropathy, cardiovascular disease, and neuropathy, which predispose them to operative complications. Additionally, they have an increased risk of surgical site infections and other infections, further increasing their likelihood of postoperative complications. 

### 6.1. Cardiovascular Disease 

Cardiovascular disease is a major risk factor for operative morbidities and mortality. Data from the Agency for Healthcare Research and Quality (AHRQ) Healthcare Cost and Utilization Project’s Nationwide Inpatient Sample showed that microvascular and coronary artery disease are associated with higher rates of mortality, myocardial infarction, and stroke in non-cardiac surgeries [49]. Individuals with diabetes are 2–4 times more likely to develop cardiovascular disease compared to those without diabetes. The relative risk for cardiovascular disease morbidity and mortality in adults with diabetes ranges from one to three in men and two to five in women [50]. A meta-analysis of 37 cohort studies reported that cardiovascular-related mortality is significantly higher in diabetics compared to the normoglycemic population (5.4% vs. 1.6%, respectively) [51]. Another large study involving 698,782 people found that diabetic patients have double the risk of developing coronary heart disease and ischemic stroke [52].

It is important to recognize that several cancer treatments—including conventional chemotherapeutic agents, novel targeted drugs, immunotherapies, and radiation therapy—can cause various cardiac toxicities [53]. A large population-based study showed that cancer patients have higher cardiovascular disease-related mortality compared to the general population [54]. Another study found higher mortality (9% vs. 2.5%) and morbidity rates (58.8% vs. 21%) in patients with non-small-cell lung cancer who also had underlying cardiovascular disease [55]. Therefore, it is imperative that diabetic cancer patients with underlying cardiovascular disease or at high risk of cardiac disease achieve optimal cardiac condition before undergoing elective surgery.

### 6.2. Nephropathy

Diabetic nephropathy is considered one of the major factors leading to end-stage renal failure worldwide [56]. Furthermore, cancer patients face a high risk of kidney injury. For example, a prospective study revealed that cancer patients with diabetes had a higher incidence of acute kidney injury (AKI), leading to significantly elevated mortality rates (15.9% vs. 2.7%) compared to those with normal renal function [57]. In a national Canadian cohort study, AKI occurred in 9.3% of oncological patients undergoing systemic chemotherapy, with myeloma patients being at the highest risk. The presence of chronic kidney disease (CKD), diabetes mellitus, or congestive heart failure was associated with AKI development [58].

The presence of impaired renal function significantly increases a patient’s risk of surgical complications. For example, a cohort study examining postoperative outcomes after hip arthroplasty in diabetic patients with or without CKD revealed a significantly higher 30-day incidence of myocardial infarction, pneumonia, sepsis, deep vein thrombosis, and pulmonary embolism in patients with both diabetes and CKD compared to those with diabetes alone [59]. In a large national clinical dataset, the development of AKI was associated with an eightfold increase in mortality. Moreover, either diabetes or underlying renal impairment was strongly associated with the development of postoperative AKI [60]. A meta-analysis of 31 cohort studies across seven countries, involving 153,885 patients, showed that patients with CKD had significantly higher rates of postoperative mortality (pooled incidence of 9.2% vs. 3.2%, respectively) and cardiovascular complications (0.8–7.6% vs. 0–28.6%, respectively) following non-cardiac procedures compared to patients with normal kidney function [61]. Therefore, the close monitoring of renal function, adequate hydration, and avoiding nephrotoxic agents in cancer patients with diabetes undergoing surgery are crucial.

### 6.3. Neuropathy

Gastroparesis, the most common manifestation of digestive autonomic neuropathy, has varying risks depending on the type of diabetes. A 10-year cohort study reported a 5.2% risk in patients with type 1 diabetes, 1.0% in those with type 2 diabetes, and 0.2% in controls [62]. Gastroparesis causes delayed gastric emptying, increasing the risk of perioperative complications such as aspiration pneumonia, acute respiratory distress syndrome, and respiratory failure [63,64].

In cancer patients, several factors may contribute to the development of gastroparesis, including microbiota disruption, heightened susceptibility to gastrointestinal infections—primarily viral—due to immunocompromised states and chemotherapy-induced neuropathy [65,66]. For gastric cancer surgeries, preoperative outflow tract obstruction and Billroth II anastomosis have been identified as risk factors for post-surgical gastroparesis [67].

Another potential complication in diabetic patients is fecal incontinence, which can result from autonomic neuropathy affecting the anal canal or chronic diarrhea [68]. Diabetic patients with rectal cancer undergoing resection face an increased risk of developing fecal and urinary incontinence, influenced by additional factors such as radiation therapy and the type and extent of surgical resection [69].

### 6.4. Infection and Surgical Wound Complications

Hyperglycemia is believed to negatively impact the immune system, increasing the risk of infections in diabetics patients. Both humoral and innate immune responses are compromised through various mechanisms, weakening the immune system [70]. These mechanisms include impaired phagocytosis, altered intercellular adhesion molecules, dysfunctional antigen presentation, neutrophil and monocyte dysfunction, the generation of oxygen free radicals, impaired growth factors, a proinflammatory state, and cytokine dysregulation [70,71]. Furthermore, cancer patients with diabetes are at high risk of neutropenia. A meta-analysis of ten observational studies, involving a total of 8688 patients, revealed that individuals with both cancer and diabetes or hyperglycemia had a higher probability of experiencing chemotherapy-induced neutropenia (odds ratio (OR) 1.32, 95% CI 1.06–1.64) compared to those without diabetes [72]. Patients with neutropenia are especially at high risk of mortality following emergency surgery [73]. 

Diabetic patients with elevated blood glucose levels in the perioperative period who underwent non-cardiac surgeries experienced significantly longer hospital and intensive care unit stays and a higher risk of infections. These infections included pneumonia (12.1% vs. 5.4%), wound and skin infections (5% vs. 2.3%), systemic blood infections (3.6% vs. 1.1%), and urinary tract infections (4.5% vs. 1.4%) [74]. A meta-analysis of 14 prospective cohort studies involving 91,094 participants found that diabetic patients were nearly twice as likely to develop surgical site infections as those without diabetes [4,75]. Higher blood glucose levels during the first postoperative day significantly increased the risk of postoperative infection [76]. There are conflicting data regarding the cutoff levels of HbA1c or blood sugar that put diabetic patients at risk of infection. For instance, higher rates of postoperative sternal wound infection (10.7% vs. 3.3%) and mediastinitis (3.8% vs. 1.3%) were observed in diabetics patients with preoperative HbA1c > 7% in cardiac surgeries [77]. Additionally, surgical site infections were more common in complicated diabetic and non-diabetic patients with neuropathy [78]. A meta-analysis examining the link between hyperglycemia and surgical site infections found that maintaining blood glucose levels below 8.3 mmol/L can reduce the risk of these complications [8,79].

The tumor location further aggravates the risk of infection. In head and neck cancer surgeries, prior irradiation and diabetes were associated with higher rates of postoperative site infections [80]. Another study reported higher rates of local complications and flap failures in diabetic patients undergoing head and neck cancer surgery [81]. A nationwide registry involving head and neck cancers found that diabetes was linked to higher postoperative infections and longer hospital stays, although it did not affect wound healing [82]. Among patients with colon cancer, those with elevated preoperative HbA1c levels and higher BMIs had a significantly higher risk of surgical site infections (59% vs. 8%; *p* < 0.001) [83]. A large retrospective cohort study of 11,633 patients who underwent elective colorectal and bariatric surgery found that perioperative hyperglycemia was associated with a twofold higher risk of postoperative infection, irrespective of pre-existing diabetes [8,84]. Similarly, Tan and colleagues reported higher postoperative complications in diabetics after colorectal surgeries, including significantly higher rates of anastomotic leakage (OR, 2.40), surgical site infection (OR, 1.98), urinary complications (OR, 1.69), and re-admission rates (OR, 1.41) [44]. Diabetes has also been linked to significantly higher rates of urinary tract infection (UTI) in patients undergoing radical cystectomy, with more than half of the patients with fungal UTIs being diabetic as per the Mayo Clinic Cystectomy Registry [85]. Additionally, radiation therapy, commonly used in various cancer treatments, can significantly impair wound healing. The long-term effects of radiation include skin atrophy, soft tissue fibrosis, and microvascular damage, leading to a higher risk of non-healing wounds [86].

Anastomotic leak is one of the severe complications of colorectal surgeries, associated with high morbidity and mortality risks [87]. A meta-analysis of 34 nonrandomized studies showed that anastomotic leak in colorectal cancer was associated with poorer overall and disease-free survival, and a higher recurrence rate [88]. A prospective study by Ziegler examined the rates of anastomotic leakage and early postoperative mortality in diabetic patients undergoing colonic resection. The study found that preoperative steroid use, regardless of diabetic status, was associated with an increased risk of anastomotic leakage. Additionally, mortality was nearly six times higher in diabetic patients with leakage compared to those without leakage (26.3% vs. 4.5%; *p* < 0.001) [89]. Another meta-analysis by Lin and colleagues identified diabetes mellitus as an independent factor for the occurrence of anastomotic leakage in colorectal surgeries (OR, 1.57, 95% CI = 1.11–2.22) [90].

Taken together, underlying diabetes and hyperglycemia significantly increase the risk of postoperative infections and surgical wound complications in various cancers. Achieving optimal blood glucose control is important to minimize these risks and improve surgical outcomes. 

## 7. Perioperative Management of Patients with Diabetes and Cancer

The outcomes of diabetic cancer patients undergoing surgery are influenced by factors related to the type of surgery, anesthesia, cancer, diabetes, and the treatment being administered (Table 1). The optimal management of diabetic patients with cancer during the perioperative period can be challenging, as the capacity to control blood glucose levels is complicated by various factors. These include nil per os (NPO) status, procedure-induced stress, recent cancer treatment, postoperative infection, and inadequate postoperative oral intake [91]. Therefore, a multidisciplinary team approach and patient education are integral components for achieving the best possible outcomes and reducing surgical complications.

The perioperative management of diabetic patients with cancer begins with a comprehensive approach involving a multidisciplinary team, including diabetologists, oncologists, surgeons, anesthesiologists, nutritionists, pharmacists, nurses, diabetes educators, and primary care physicians. It is important for team members to collaborate in developing individualized care plans tailored to each patient’s unique needs. Additionally, patient education is a cornerstone of this approach that empowers patients to actively participate in their care, self-management, and adherence to treatment plans. By providing education on glycemic control, medication adherence, preoperative preparation, postoperative care, lifestyle modifications, and available support resources, patients are better equipped to navigate their surgery and achieve optimal outcomes (Table 2).

### 7.1. Preoperative Optimization 

Evidence regarding the perioperative management of diabetes is regrettably scarce. Consequently, many guidelines are based on expert opinion and best practice panel consensus [8,92]—most guidelines do not specifically address the management of cancer patients. The recommended target glucose level for cancer patients with diabetes is 6.0–10.0 mmol/L, and an acceptable range is defined as 6.0–12.0 mmol/L [93,94]. However, less stringent control may be appropriate for individuals at high risk of hypoglycemia, those with a poor prognosis, or those receiving supportive care and requiring surgical procedures. 

The United Kingdom-based Centre for Perioperative Care (CPOC) Guideline for Perioperative Care for People with Diabetes Mellitus Undergoing Elective and Emergency Surgery recommends referral to a preoperative assessment center for all patients with diabetes who are undergoing elective surgery that requires fasting [95]. In contrast, the American Diabetes Association (ADA) Standards of Care 2024 Guidelines suggest limiting such a referral to diabetic patients with autonomic neuropathy or renal failure, or who are at risk of ischemic heart disease. We recommend the preoperative assessment of all patients with both cancer and diabetes who are required to fast.

The first step in the preoperative assessment of diabetes is to determine the diabetes type [8]; approximately 90% of diabetic patients have type 2 diabetes [7]. However, it is essential to identify patients with type 1 diabetes, as they will require either basal insulin or an IV insulin infusion to reduce the risk of diabetic ketoacidosis due to their absolute insulin deficiency [8,95]. It is also important to optimize existing diabetic complications such as nephropathy, autonomic neuropathy, coronary heart disease, peripheral vascular disease, and hypertension. Additionally, each patient’s preoperative diabetes management should be reviewed, focusing on the frequency of blood glucose monitoring, average blood glucose levels, HbA1c levels, and any prior instances of hypoglycemia. A detailed history of each patient’s diabetes treatment, particularly their use of medications that can cause hypoglycemia such as insulin, sulfonylureas, and meglitinides, should be assessed. The type and timing of cancer treatments and other concurrent medications must also be reviewed. Important factors to consider include the type of planned surgery (oncologic or non-oncologic), the required fasting period, the duration of the procedure, the use of perioperative steroids, and the feasibility of continuing insulin infusion devices during surgery. Finally, the type of anesthetic planned—whether epidural, regional, or general—should be considered, as epidural and regional anesthesia have minimal effects on glucose metabolism and insulin resistance.

HbA1c should be measured within three months of elective surgery [8]. Generally, an HbA1c target of less than 8% is recommended for elective procedures [92]. A post hoc analysis of the Surgical Site Infection Trial demonstrated that among patients who self-reported not having diabetes, 65% had HbA1c levels above 6%. This subgroup also had the highest infection rate of 39% after major surgery [8,96]. These findings underscore the importance of evaluating HbA1c levels in cancer patients without prior diabetes diagnosis before surgery. Patients with blood glucose levels above 11 mmol/L due to poor diabetes control, steroid use, or targeted therapy causing hyperglycemia require insulin to optimize blood glucose before surgery.

Measures to improve the postoperative outcomes of diabetic patients include the use of minimally invasive surgery where appropriate, which has been shown to result in lower postoperative complications and shorter hospital stays [97]. Patients on active chemotherapy or targeted therapy, including anti-angiogenesis agents, should cease treatment 4–6 weeks prior to surgery to avoid complications such as impaired wound healing, bleeding, and infection [98]. Other anticancer medications—such as hormone therapy—that do not increase the risk of cytopenia, metabolic abnormalities, bleeding, or impaired wound healing, can be continued. Surgeries for such patients should be scheduled to take place in the early morning to minimize the disruption of blood glucose management while they are nil per oral. Optimizing nutrition and physical conditioning are equally important for achieving optimal surgical outcomes. 

### 7.2. Perioperative Management of Blood Glucose

Maintaining normoglycemia during surgery and the avoidance of marked hyperglycemia, hypoglycemia, and ketoacidosis is paramount for diabetic cancer patients. Continuous monitoring of blood glucose levels is essential to allow for timely adjustments in insulin therapy, thereby preventing both hyperglycemia and hypoglycemia. Tight glycemic control, which is defined as maintaining blood glucose levels between 4.4 and 6.1 mmol/L, has been a subject of considerable research and debate.

Early studies conducted in critically ill patients demonstrated that intensive insulin therapy to maintain tight glycemic control resulted in better mortality and morbidity rates compared to the standard glucose target of 10–11.1 mmol/L [99]. However, a recent update of a Cochrane meta-analysis of 20 randomized controlled trials found that intensive glycemic control made little to no difference in all-cause mortality compared to conventional glycemic control in people with diabetes undergoing surgery (relative risk = 1.08, 95% CI = 0.88 to 1.33) [100]. Although intensive insulin therapy could improve certain outcomes, the higher incidence of hypoglycemia could negate these benefits [101]. Hypoglycemia is particularly detrimental in surgical patients and critically ill patients, as it can lead to adverse neurological outcomes, cardiac arrhythmias, and even increased mortality. Therefore, a careful and individualized approach to managing blood glucose levels in diabetic cancer patients during surgery is essential. The goal should be to maintain stable and safe glucose levels, aiming for moderate glycemic targets that minimize the risk of hypoglycemia while still providing the benefits of controlled blood glucose levels. This involves continuous glucose monitoring, tailored insulin regimens, and a multidisciplinary team to manage the complex interplay of factors affecting glucose metabolism during surgery. 

Patients on parenteral nutrition (TPN) often experience elevated blood glucose levels that necessitate appropriate management with insulin. Initial management may involve a variable-rate insulin infusion accompanied by a dextrose infusion, transitioning to adding 80–100% of the total insulin requirement directly to the TPN solution. The daily insulin requirement can be established using a subcutaneous sliding scale. Alternatively, twice-daily doses of intermediate insulin can be administered with TPN. If TPN is interrupted for over an hour, IV dextrose and insulin recalibration are needed. Similarly, continuous enteral nutrition requires careful insulin management and coordination among healthcare providers.

### 7.3. Perioperative Management of Oral and Non-Insulin-Injectable Diabetes Medication 

A key step in perioperative diabetes control is that of managing medications, including oral and injectable non-insulin antihyperglycemics and insulin. It is recommended to withhold oral antihyperglycemics on the morning of surgery, with the exception of sodium–glucose co-transporter 2 inhibitors (SGLT2i) such as empagliflozin, dapagliflozin, canagliflozin, which should be held 3 days prior to surgery (4 days for ertugliflozin) due to the risk of ketoacidosis post-surgery [92,102,103]. SGLT2i can be restarted once the patient has stable renal function and is adequately hydrated [7]. It is recommended to withhold metformin on the day of surgery [92]; it can be restarted after 24 h if the patient has stable renal function. Sulfonylureas and meglitinides, which are oral insulin secretagogues, carry an inherent risk of causing hypoglycemia [7,92]. It is recommended to withhold sulfonylureas, meglitinides, dipeptidyl peptidase IV inhibitors (DPP4i), thiazolidinediones, and alpha-glucosidase inhibitors on the morning of surgery and to restart them once the patient resumes eating [92]. There is limited evidence regarding the perioperative use of glucagon-like peptide (GLP)-1 receptor agonists such as liraglutide and semaglutide [92]. These medications are increasingly being used for diabetes and weight loss, but concerns exist about delayed gastric emptying and aspiration risk [7,103,104]. However, a recent report did not confirm an increased risk of respiratory complication with the preoperative use of GLP-1 receptor agonists [105]. Consensus recommendations suggest withholding short-acting GLP-1 receptor agonists for one day and long-acting formulations for one week before surgery [103]. For patients taking GLP-1 receptor agonists for weight loss, it is recommended to hold semaglutide for three weeks [104]. 

### 7.4. Perioperative Management of Insulin

For patients with type 1 diabetes or insulin-dependent type 2 diabetes, maintaining basal insulin is essential to prevent diabetic ketoacidosis and limit protein loss during periods of reduced caloric intake and perioperative stress. Basal insulin typically constitutes about half of the total daily insulin dose. The ADA recommends reducing long-acting insulin to 75–80% of the usual dose on the morning before surgery or reducing the basal dose by 25% the evening before surgery [92]. Additionally, it is recommended to halve the NPH insulin dose on the morning of surgery. For patients using premixed insulin two or three times per day, it is recommended to administer 50% of the usual dose on the day of surgery [92]. Patients using an insulin pump can typically continue their usual basal infusion rate as long as the pump can be safely used during the surgical procedure; if use of the pump needs to be discontinued prior to surgery, then patients should receive basal insulin according to their programmed settings 2–3 h before discontinuing pump usage. Maintaining glucose levels between 5 and 10 mmol/L in a fasting state is appropriate [8]. In those patients undergoing non-cardiac general surgery, the use of basal bolus insulin regimens when they resume eating is associated with improved glycemic outcomes and lower perioperative complications compared to reactive sliding-scale insulin regimens [92].

### 7.5. Postoperative Management

Postoperative care for cancer patients with diabetes is centered on maintaining glycemic control to reduce infection risks and facilitate wound healing. Standard practices include the monitoring of blood glucose levels and the use of sliding scales for insulin dosing as necessary, particularly in the immediate postoperative period when patients are not yet feeding.

In addition to glycemic management, postoperative care for these patients involves other crucial aspects such as nutritional support and early mobilization. Adequate nutrition is vital for promoting healing and preventing complications, whereas early mobilization helps to prevent issues such as deep vein thrombosis and pulmonary embolism, which are of particular concern in surgical patients with cancer. 

Continuous collaboration among healthcare providers during the postoperative phase is important for comprehensive management and achieving optimal outcomes for diabetic cancer patients undergoing surgery. Moreover, the timing of anticancer systemic therapy, such as anti-VEGF agents, requires careful consideration in postoperative care. Typically, these therapies can be initiated four weeks after surgery if no postoperative complications, such as surgical site infections, arise.

## 8. Future Directions

The perioperative management of patients with both cancer and diabetes is an area in which high-quality evidence is limited. Most current guidelines and recommendations rely on consensus and expert opinions rather than robust clinical evidence, and they are not specifically tailored to cancer patients. Therefore, future prospective studies are needed to better inform the perioperative management of cancer patients with diabetes, particularly regarding the effects of surgical complications on subsequent cancer therapy and outcomes. Novel pharmacological targets that could better control diabetic complications and potentially cure diabetes are currently being explored [106]. In addition, the potential role of wearable technology in monitoring and managing diabetes in cancer patients during the perioperative period is an emerging area that warrants further exploration. 

There are a few prospective studies currently underway, and most do not focus on cancer patients (Table 3). 

The Management and Outcomes of Perioperative Care among European Diabetic Patients (MOPED) study (NCT04511312) is an international, prospective, observational cohort study involving diabetic patients undergoing either elective or emergency surgery [107]. The primary endpoint of this study is the number of days patients spend at home within 30 days post-surgery. Other key endpoints include the Comprehensive Complications Index, postoperative Quality of Recovery score, 30-day mortality, length of hospital stay, incidence of major adverse events, time to resume normal diabetes therapy, occurrences of diabetic ketoacidosis or hypoglycemia, use of intravenous insulin infusion, and changes in diabetes management at 30 days. Although the study does not primarily focus on cancer patients, a subgroup analysis of cancer patients with diabetes will provide new prospective data on preoperative diabetic management in this group.

NCT06314061 is a randomized controlled trial investigating the effect of continuous glucose monitoring compared to standard point-of-care blood glucose measurements in surgical patients with diabetes. This trial could provide real-time data and potentially improve outcomes by allowing for more precise and timely adjustments in the management strategies of cancer patients with diabetes.

## 9. Summary

Both diabetes and cancer are rapidly growing global health issues that are among the leading causes of morbidity and mortality. The relationship between these two conditions is complex and bidirectional. Many studies have shown that cancer patients have a higher risk of developing diabetes, and conversely, that diabetic patients are at an increased risk of certain cancers. Additionally, the data indicate that cancer patients with diabetes experience poorer survival outcomes and more severe therapy-related complications compared to the general population. Many individuals who are diagnosed with cancer will undergo some type of surgical procedure; cancer patients with diabetes have elevated risks of surgical complications and postoperative mortality, making optimal blood glucose control crucial. Diabetic patients often have several underlying comorbidities such as nephropathy, cardiovascular disease, and neuropathy that predispose them to operative complications. Furthermore, the type of surgery, patient-related factors, underlying cancer, and its treatment could influence the surgical outcomes of these patients.

The perioperative management of diabetic patients with cancer begins with a comprehensive approach involving a multidisciplinary team. For elective surgeries, the recommended HbA1c target is below 8%, and the target glucose level is 6.0–10.0 mmol/L, alongside an acceptable range of 6.0–12.0 mmol/L. In addition to glycemic management, key aspects of perioperative care necessary for optimal outcomes in these patients include managing comorbid illnesses, adjusting diabetes and cancer treatments perioperatively, optimizing nutrition, reducing fasting duration, promoting early mobilization, delivering patient education, and supporting self-management. Currently, comprehensive data on the perioperative management of patients with both diabetes and cancer are lacking. Future studies are needed to develop more effective therapies to improve overall outcomes for these patients.

## Figures and Tables

**Table 1:** Factors influencing surgical outcomes in patients with diabetes and cancer.

Factors	Comments
Surgery-Related Factors	
Type of surgery	Surgical approach and invasiveness influence perioperative risk and recovery.
Emergency vs. elective	Emergent surgeries may carry higher risks and require immediate attention to perioperative management.
Curative vs. palliative	The distinction between curative and palliative surgery guides treatment goals and long-term management strategies.
Patient-Related Factors	
Age	Increased age can lead to higher surgical risk due to frailty and comorbidities.
Body mass index (BMI)	Higher BMI is associated with increased surgical complications such as infections and delayed wound healing.
Comorbid illness	Comorbidities can complicate anesthesia and postoperative recovery.
Functional and nutritional status	Poor functional and nutritional status may predict poor surgical outcomes and delayed recovery.
Psychosocial factors	Social support, mental health status, and coping mechanisms impact postoperative recovery.
Cancer-Related Factors	
Type of cancer	Different cancers may have varying impacts on surgical risk and recovery.
Stage of the cancer	Advanced stages can increase surgical complexity and risk.
Disease burden in advanced cancer	High disease burden can lead to increased surgical risk and complications.
Diabetes-Related Factors	
Type of diabetes	Type 1 vs. type 2 diabetes may influence perioperative glucose management strategies.
Duration	Longer duration of diabetes can lead to more complications such as cardiovascular disease and nephropathy, affecting surgery.
Control	Poorly controlled diabetes increases the risk of infections, poor wound healing, and other complications.
Insulin vs. oral hypoglycemic medication	Insulin-dependent patients may have more complex perioperative management compared to those on oral medications.
Cancer Treatment-Related Factors	
Timing of treatment	Recent treatments can affect immune response and healing capacity.
Type of systemic therapy	Chemotherapy, targeted therapy, and immunotherapy have different impacts on surgical risk and recovery.
Tendency to cause hyperglycemia	Some treatments, especially steroids, can increase blood glucose levels, complicating perioperative management.

**Table 2:** Perioperative management of diabetes in cancer patients.

Intervention	Recommendation	Comments
Glucose target level	6.0–10.0 mmol/L	A less stringent control will be appropriate for individuals with risk of hypoglycemia
HbA1c target	<8%	
Optimize concurrent illnesses		Includes hypertension, cardiovascular diseases, and renal function
Duration of fasting	Minimize preoperative fasting time	IV dextrose is recommended with basal insulin
Nutrition	Provide adequate nutrition and manage enteral/parenteral feeding	Nutritional support should be tailored to individual patient needs
Patient’s education	Educate patients on diabetes management during the perioperative period	Include information on medication adjustments, monitoring blood glucose, and recognizing signs of hypo/hyperglycemia
Cancer treatment		
Chemotherapy	Stop 4 weeks before surgery	Coordination with oncology team
Targeted therapy	Stop anti-VEGF (e.g., bevacizumab) 4–6 weeks before surgery; most other targeted therapies can be stopped 2 weeks before surgery	
Diabetic medication		
Non-insulin diabetic medication	Stop SGLT2 3–4 days before surgery	Includes canagliflozin, dapagliflozin, and empagliflozin
Short-acting GLP-1 receptor agonists	Short acting for one day before surgery	Includes exenatide and lixisenatide
Long-acting GLP-1 receptor agonists	Stop one week before surgery	Includes dulaglutide and semaglutide
All other oral medication held on surgery	Hold on the day of surgery	Includes metformin, sulfonylureas, DPP-4 inhibitors, etc.
Long-acting insulin	Administer 75–80% of the usual dose on the morning of surgery	
Premixed insulin two or three times per day	Administer 50% of the usual dose on the day of surgery	
NPH insulin dose	50% of the usual dose on the day of surgery	
Feeding and mobilization	Initiate feeding and mobilization as soon as clinically feasible post-surgery	Early feeding and mobilization can help in faster recovery and better glucose control
Self-management	Provide resources and support for self-management post-discharge	Follow-up appointments and ongoing diabetes education

**Table 3:** Ongoing study in the perioperative management of diabetes with or without cancer.

ID	Type	Title	Sample Size	Location	Time Period
NCT04511312	Prospective observational study	Management and Outcomes of Perioperative Care Among European Diabetic Patients: (MOPED): A Prospective Observational, International Cohort Study	5000	Europe	2021–2024
NCT06295289	Open-label, randomized controlled trial	Hybrid Closed-loop Insulin Delivery System in Perioperative Diabetic Patients: an Open-label, Randomized Controlled Trial	54	China	2023–2024
NCT05547594	Prospective observational study	Impact of a Multimodal Prehabilitation Programme on Markers of Health, Quality of Life and the Short and Long Post-surgery Recovery Period in Cancer Patients With Type 2 Diabetes	60	United Kingdom	2023–2024
NCT06314061	Randomized controlled trial	The Effect of Continuous Glucose Monitoring With Real-time Alerts on Glycaemic Control in Surgical Patients With Diabetes: A Randomised, Clinical Multicentre Trial	200	Denmark	2024–2026
NCT03945968	Prospective observational study	The Role of Concomitant Diseases in Postoperative Complications Risk Stratification (STOPRISK)	16,000	Russia	2019–2024
